# Celastrol delays hepatic steatosis and carcinogenesis in a rapid AKT/c-Met-transfected hepatocellular carcinoma model *via* suppressing fatty acid synthase expression and AKT/ERK phosphorylation†

**DOI:** 10.1039/c8ra00522b

**Published:** 2018-04-16

**Authors:** Junjie Hu, Xin Li, Junxuan Zhou, Cong Zhang, Guohua Zheng, Zhenpeng Qiu

**Affiliations:** College of Pharmacy, Hubei University of Chinese Medicine No. 1, West Huangjiahu Road Wuhan 430065 People’s Republic of China whuqq@hotmail.com; Key Laboratory of Chinese Medicine Resource and Compound Prescription, Ministry of Education, Hubei University of Chinese Medicine No. 1, West Huangjiahu Road Wuhan 430065 People’s Republic of China zgh1227@sina.com

## Abstract

Although the suppressing effects of celastrol on hepatocellular carcinoma (HCC) have been demonstrated, evidence for the targeting of fatty acid synthetase (FASN) in the development of HCC by celastrol is still rare. In this study, the effect of celastrol on a rapid HCC model featuring co-activation of AKT/c-Met oncogenes in mice was studied. The effect of celastrol on the alpha-fetoprotein level in the liver and serum was also investigated. Protein expressions of PCNA, Ki67 and FASN in celastrol-treated AKT/c-Met HCC mice were observed. The molecular mechanism of celastrol on the AKT/c-Met signaling pathway was elucidated. The results revealed that celastrol significantly repressed the AKT/c-Met induced HCC development and down-regulated the mRNA expression of AFP in the liver and the AFP level in serum. Furthermore, the expression of proliferation-associated proteins in the HCC tissues was reduced by celastrol treatment. Moreover, the abundant steatosis that resulted from FASN accumulation in the liver in AKT/c-Met mice was also attenuated. Finally, the phosphorylation of AKT and ERK1/2 in HCC mice was repressed by celastrol treatment. Overall, our data demonstrate that celastrol exerts an antiproliferative and novel lipid-decreasing effect by targeting AKT/ERK and FASN in HCC development *in vivo*.

## Abstract 2

In this study, the effect of celastrol on a rapid HCC model featuring co-activation of AKT/c-Met oncogenes in mice was studied.

## Graphical abstract

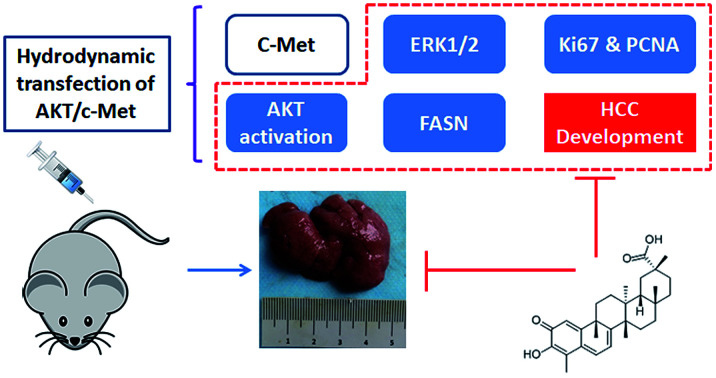

## Introduction

Hepatocellular carcinoma (HCC) is the most common primary liver cancer, and it has a rapidly rising mortality worldwide.^1^ Infiltrating proliferation, intrahepatic metastasis and high deterioration are characteristic of HCC. HCC patients are commonly diagnosed at advanced stages where surgical resections are unsuitable. Although radiotherapy, chemotherapy and some novel multikinase inhibitor (*e.g.*, sorafenib) based treatments for targeting abnormal proliferation and angiogenesis are considered as the standard strategies for advanced HCC, their effect on increasing the survival rate is still unsatisfactory.^2^ Therefore, the discovery of novel therapeutic agents from other available resources is necessary for treating HCC.

Serine/threonine phosphorylation of v-akt murine thymoma viral oncogene homolog (AKT) triggered by activation of phosphoinositide-3 kinase (PI3K) regulates a nutrient/energy/redox sensor and controller that mobilizes the oncogenic protein synthesis, mTORC1.^3^ Moreover, mTORC1 is the modulator for suppressing 4-EBP1/eIF4E signaling or activating p70S6K/RPS6 to block cell apoptosis or promote cell proliferation, respectively.^4^ Furthermore, the PI3K/AKT signaling pathway is a kind of key cascade frequently activated in hepatic tumorigenesis. Hence, de-phosphorylation of AKT by small molecule compounds could be effective for inhibiting liver cancer development and progression. The c-Met carcinogenic gene encodes the high-affinity receptor tyrosine kinase binding hepatocyte growth factor (HGF) to achieve auto-phosphorylation. The active HGF/c-Met signaling pathway in an aggressive growth enhances cell survival, invasion and metastasis,^5^ and is positively correlated with a bad prognosis of HCC.^6^ In this respect, c-Met might be synergistic with accelerating PI3K/AKT-induced hepatic malignancy and could be an available therapeutic target for HCC remedies.

Celastrol is a triterpene quinine methide isolated from the root bark of *Tripterygium wilfordii* Hook F., possessing multiple biological functions, notably including inflammation and carcinoma suppression. Previous studies have confirmed that celastrol could obstruct nuclear transportation of nuclear factor κB (NF-κB), which could inhibit the inflammatory response in LPS-stimulated RAW264.7 cells.^7^ Moreover, celastrol exerts the aforementioned function to repress tumorigenesis, tumor invasion and metastasis on various *in vitro* oncocytes and *in vivo* neoplasm models by targeting proteasomes, VEGFR expression, pro-oncogenic microRNA and AKT/mTOR & MAPK pathways.^8^ Although the effects of celastrol on carcinogenesis such as suppressing E2F1 (ref. 9) or microRNA-224,^10^ modulating the STAT3/JAK2 cascade^11^ and triggering ER stress^12^ have been reported previously in some HCC models, the molecular mechanism of celastrol on HCC development has still not been precisely documented. In this work, based on a novel HCC mouse model *via* hydrodynamic transfection featuring overexpression of AKT and c-Met oncogenes and excessive lipid accumulation in the liver, the effects of celastrol on hepatic steatosis and carcinogenesis have been demonstrated. This study provides the first evidence that celastrol is effective in alleviating liver steatosis and de-phosphorylating AKT/extracellular signal-regulated kinase (ERK) in the development process of HCC.

## Materials and methods

### Constructs and reagents

Celastrol was purchased from Sigma-Aldrich (St. Louis, MO, USA). The plasmids, including pT3-EF1α-HA-myr-AKT, pT3-EF1α-V5-c-Met and pCMV/sleeping beauty transposase (SB), used for mouse hydrodynamic transfection, were obtained from Professor Xin Chen’s laboratory at the University of California, San Francisco (UCSF). The E.Z.N.A.® Plasmid Maxi Kit (Omega Bio-tek, Inc. Doraville, GA, USA) was used for construct purification before hydrodynamic injection in mice.

### Hydrodynamic transfection and celastrol treatment

Wild-type FVB/N mice were obtained from Charles River (Beijing, China). In a preliminary study, we administered celastrol (1, 2 and 4 mg kg^−1^) to FVB/N mice (*n* = 6, each group) to confirm the practicable doses. It was found that there was no obvious weight reduction or food intake decrease in the two dosage groups with 1 and 2 mg kg^−1^ after 3 weeks of celastrol-added administration. However, two mice died in the group with 4 mg kg^−1^ celastrol treatment on the 10th and 13th days, respectively (data not shown). Therefore, the mice received 1 and 2 mg kg^−1^ of celastrol for 3 weeks in this study. Hydrodynamic transfection was carried out as previously described.^13^ In brief, the overexpressing constructs encoding AKT (HA-tag) (20 μg) and c-Met (V5-tag) (20 μg) along with sleeping beauty transposase (SB) (1.6 μg) in a ratio of 12.5 : 12.5 : 1 were diluted in 2 mL normal saline and injected into the caudal vein of the mice in approximately 6 seconds. Celastrol (1 or 2 mg) or vehicle (70% cremophor/ethanol 3 : 1, 20% PBS, and 10% DMSO) was intraperitoneally injected daily for 3 weeks, starting 4 weeks after AKT/c-Met construct injection for early stage AKT/c-Met HCC treatment (see ESI Fig. S1†). Mice (*n* = 6, each group) were kept at the humidity-controlled temperature of 22 ± 3 °C and in an artificial light/dark cycle (12 h : 12 h). The Animal Ethics Committees of the Hubei University of Chinese Medicine approved all of the experimental protocols in accordance with the Principles of Laboratory Animal Care and Use in Research (Ministry of Health, Beijing, China). When the trial ended, the mice were sacrificed by decapitation. Any AKT/c-Met injected mice with low expression of total AKT in the liver samples were excluded from further investigation.

### Alpha-fetoprotein (AFP) ELISA assay

Serologic AFP levels in mouse serum samples collected at the end of the experimental period were assessed using a Mouse alpha-Fetoprotein/AFP Quantikine ELISA Kit (R&D System Inc., Minneapolis, MN) according to the manufacturer’s instructions.

### Hematoxylin and eosin (H&E) staining and immunohistochemistry

Liver samples obtained from the mice were immersed in 4% paraformaldehyde and embedded in paraffin. For H&E staining, liver tissue sections were dewaxed in xylene and rehydrated using ethanol with decreasing concentrations. The sections were then stained with hematoxylin and eosin and dehydrated using ethanol and xylene.

For immunohistochemistry, de-paraffinized sections were immersed in phosphate-buffered saline (PBS) solution with 3% H_2_O_2_ for 30 minutes to eliminate the activity of the endogenous peroxidase. Then slides were microwaved in 10 mM citrate buffer (pH 6.0) for 15 minutes to retrieve antigens. Slides were further incubated with Ki67 (CST, USA) and FASN (CST, USA) primary antibodies for 16 h at 4 °C. After incubation with the appropriate biotin-conjugated secondary antibody and subsequently with streptavidin solution, color development was performed using Vector NovaRED™ (Vector Laboratories) as a chromogen. Sections were counterstained using Gill-2 hematoxylin (Thermo-Shandon, Pittsburgh, PA, USA).

### Western blotting

Proteins in the mouse liver specimens were extracted using Mammalian Protein Extraction Reagent (Thermo Scientific, Waltham, MA) containing Cocktail Protease Inhibitor (Roche, Indianapolis, IN, USA). A BCA protein determination assay was performed for quantification of the protein concentration. The lysates were denatured at 95 °C in Tris–glycine SDS loading buffer. After the gradient was separated using SDS-PAGE and blotted to polyvinylidene fluoride membranes, the membranes were blocked, and the proteins on the membranes were probed with specific primary antibodies. The following primary antibodies (CST, USA) were used: phosphor-AKT (Thr308), phosphor-AKT (Ser473), total AKT (t-AKT), phospho-S6 ribosomal protein (Ser235/236), PKM2 (D78A4), phospho-p44/42 MAPK (ERK1/2) (Thr202/Tyr204), and total ERK1/2 (t-ERK1/2). Each protein linked with primary antibody was further incubated with horseradish peroxidase-secondary antibody for 1 hour at 25 °C. The protein expressions were exhibited with enhanced chemiluminescence (ECL) reagent (Thermo Scientific, USA). The HRP-conjugated β-actin antibody (Proteintech, Wuhan, China) served as an internal standard for normalization.

### Quantitative real-time polymerase chain reaction (qPCR)

The total cellular RNA in the liver samples was extracted and isolated using TRIzol® Reagent (Invitrogen, USA). About 1.0 μg of the total RNA was used as the template in the reverse–transcription reaction using ReverTra Ace (Toyobo, Japan) with an oligo (dT) 18 for generating complementary DNA (cDNA) at 37 °C for 60 min. The quantitative polymerase chain reaction (qPCR) was performed on a Mini-Opticon™ (Bio-Rad, USA) using FastStart Universal SYBR Green Master (Roche Diagnostics, Indianapolis, IN, USA). After pre-denaturation at 95 °C for 2 min, the transcriptional targets were amplified with 40 cycles at 95 °C for 5 s and 55 °C for 25 s (two-step PCR). The average Ct value of the β-actin (F: 5′-CGT TGA CAT CCG TAA AGA CCT C-3′; R: 5′-TAG GAG CCA GGG CAG TAA TCT-3′)^14^ gene was calculated for normalizing the mRNA expressions of AFP (F: 5′-TCT GCT GGC ACG CAA GAA G-3′; R: 5′-TCG GCA GGT TCT GGA AAC TG-3′).^15^

### Statistical analysis

Data analysis was performed with Prism 6 (GraphPad, USA). All data are shown as means ± SD (*n* = 5). Comparisons between 2 groups were performed with a two-tailed unpaired *t*-test. Comparisons among three or more groups were performed with ANOVA. *P* values < 0.05 were considered statistically significant.

## Results

### Rapid HCC development in mice induced by co-activation of AKT/c-Met is delayed by celastrol

**Fig. 1:**
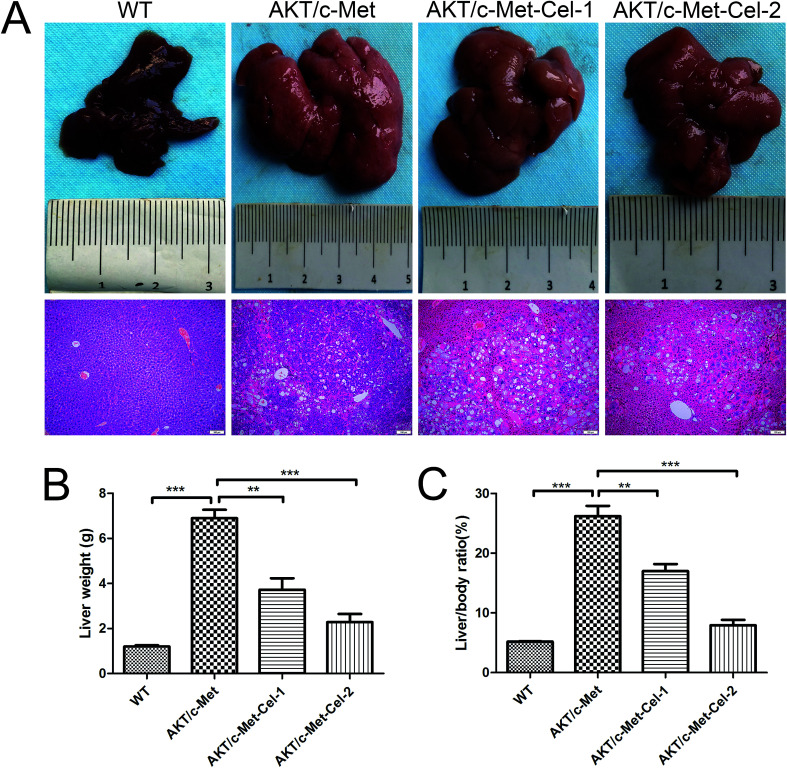
Celastrol effectively inhibits tumor growth in AKT/c-Met HCC mice. (A) Macroscopic appearance and H&E staining (scale bar: 100 μm) of AKT/c-Met mouse livers with celastrol treatment (1 or 2 mg kg^−1^) for 3 weeks. (B) The liver weight and liver/body ratio (C) of AKT/c-Met mice were both reduced by celastrol treatment. *n* = 5/group. **p < 0.01; ***p < 0.001. AKT/c-Met-Cel-1 and AKT/c-Met-Cel-2 represent tumor burden groups with 1 or 2 mg kg^−1^ celastrol, respectively.

As described in our previous study, overexpression of activated AKT combined with c-Met by hydrodynamic transfection induces rapid (6–8 weeks) hepatocarcinogenesis *in vivo*. In the present study, co-activation of AKT/c-Met resulted in liver steatosis and hepatomegaly, causing typical HCC development within 7 weeks post-injection. Based on this model, the therapeutic effects of celastrol on AKT/c-Met HCC mice were investigated. To do so, AKT/c-Met mice were intraperitoneally administered with celastrol at 1 or 2 mg per kg per day. In striking contrast, the data for liver weight and liver/body ratio suggested that the process of tumorigenesis in liver tissues from celastrol-treated AKT/c-Met mice, especially from the high dose group, appeared to be abolished (Fig. 1A). Moreover, liver steatosis, a typical histopathology in AKT/c-Met mice, was also alleviated by celastrol (Fig. 1A, H&E staining).

The small molecular multi-kinase inhibitor sorafenib has been approved by the Food and Drug Administration (FDA)^16^ as the first therapeutic strategy for the treatment of HCC based on data from the phase III Sorafenib HCC Assessment Randomized Protocol (SHARP) trial.^17^ Therefore, we employed sorafenib as the control for celastrol treatment on AKT/c-Met-induced HCC in mice. Hence, we also observe whether sorafenib could suppress HCC progression in AKT/c-Met mice. However, no changes in either liver macroscopic appearance or weight were detected in sorafenib (100 mg kg^−1^) (a kinase inhibitor approved for treating primary liver cancer) treated AKT/c-Met HCC (see ESI Fig. S2†).

### Celastrol suppresses AFP gene transcription and reduces AFP secretion in AKT/c-Met mice

**Fig. 2:**
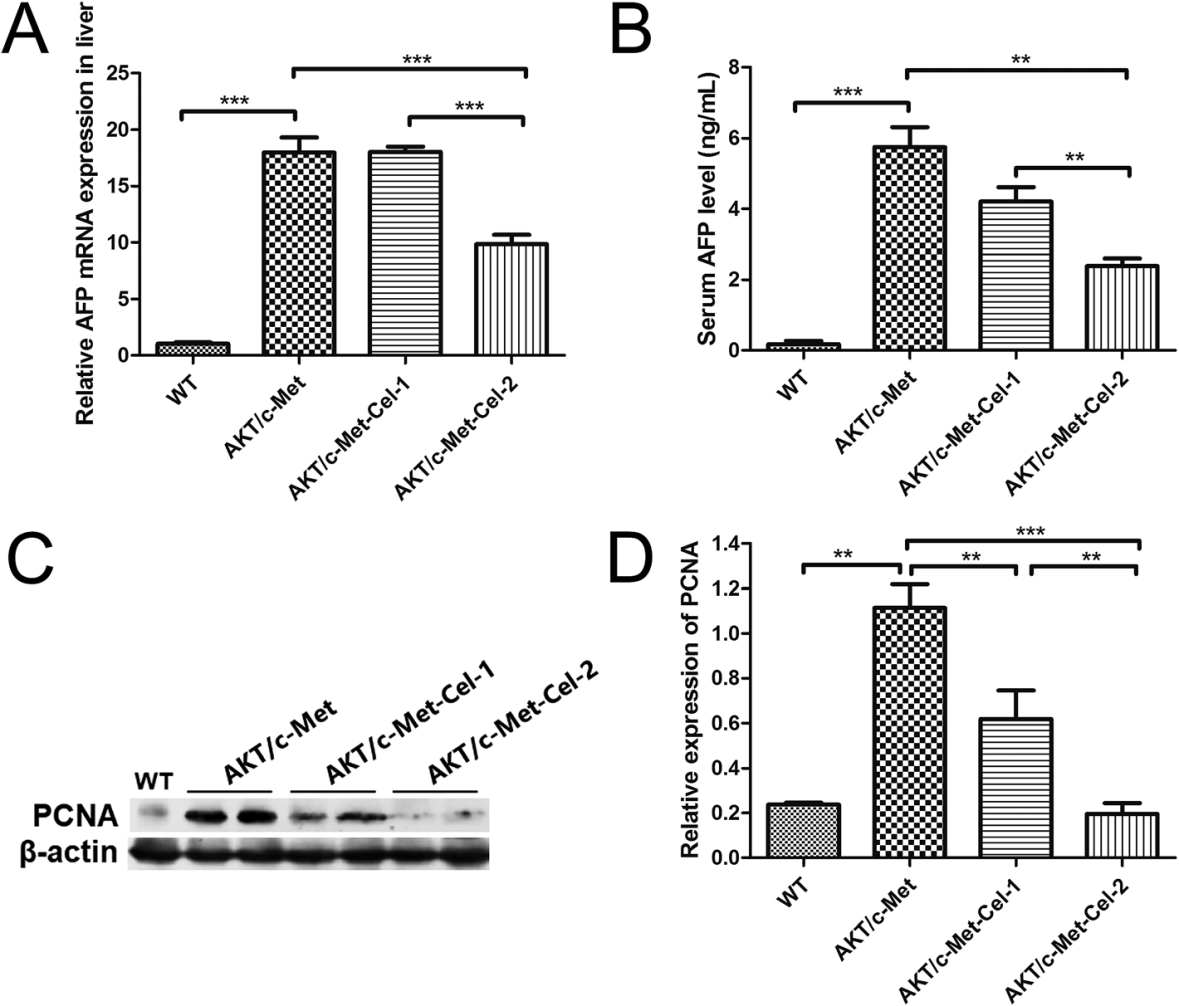
Celastrol reduces AFP secretion and suppresses the protein expression of PCNA in AKT/c-Met mice. (A) The effects of celastrol on the MRNA expression of AFP in the liver samples. The RT-qPCR assay was performed using MRNA from liver samples treated with celastrol and the protocol is described above. (B) An ELISA assay was performed to quantitate the AFP concentration in serum. AFP secretion was reduced by celastrol in the high dose (2 mg kg^−1^) group, but not in the low dose (1 mg kg^−1^) group. The values in A and B are given as the mean ± sd, *n* = 5/group. A western blot assay was performed, and representative bands are illustrated in (C). (D) Histograms indicate the relative protein expression of PCNA quantified using western blot optical analysis. The housekeeping gene β-actin was applied as an internal reference. Data are expressed as mean ± sd. *n* = 5/group. **p < 0.01; ***p < 0.001. AKT/c-Met-Cel-1 and AKT/c-Met-Cel-2 represent tumor burden groups with 1 or 2 mg kg^−1^ celastrol, respectively.

AFP is a diagnostic and prognostic HCC marker for more than 70% of primary liver cancers, and is clinically employed to grade the progression of HCC. Thus, we also investigated the therapeutic effect of celastrol on AKT/c-Met induced HCC by quantitating the mRNA expression of AFP in livers and AFP secretion in serum using qPCR and ELISA assays, respectively. The results showed that the treatment with celastrol in the high dose group (2 mg kg^−1^) showed a significant decrease in the mRNA expression of AFP (Fig. 2A). Furthermore, serum AFP levels, which were elevated in AKT/c-Met mice, were also down-regulated by celastrol (Fig. 2B). Thus, our results suggest that celastrol could reduce the serum level of the liver cancer marker AFP, which is attributable to the down-regulating effects of celastrol on the AFP mRNA transcriptional levels in mouse livers exhibiting AKT/c-Met hepatocarcinogenesis.

### Proliferation-related proteins and FASN could be suppressed by celastrol in AKT/c-Met HCC

**Fig. 3:**
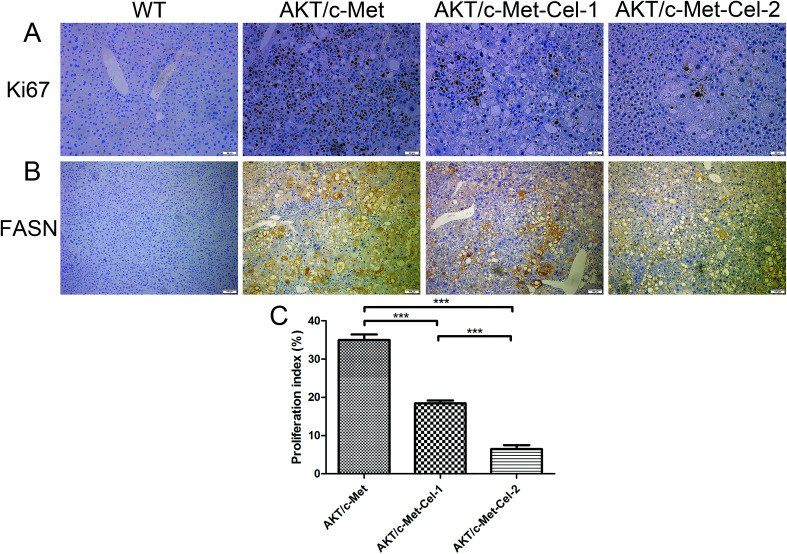
Celastrol down-regulates the expressions of nuclear antigen Ki67 and FASN. The IHC assay was performed as described above. (A) Observation of nuclear Ki67 staining in the AKT/c-Met HCC tissues. The AKT/c-Met induced high expression of Ki67 was suppressed by celastrol treatment. Original magnification: 200×. (B) Immunohistochemical patterns of FASN in the liver of AKT/c-Met HCC mice treated with celastrol. The sample from AKT/c-Met HCC mice shows the high expression of FASN. The celastrol in the high dose (2 mg kg^−1^) group partly reduces the accumulation of FASN. Original magnification: 100×. (C) The Ki-67 positive cells in (A) were counted and quantified as a proliferation index (the percentage of positive staining area of the whole area). ***p < 0.001. AKT/c-Met-Cel-1 and AKT/c-Met-Cel-2 represent tumor burden groups with 1 or 2 mg kg^−1^ celastrol, respectively.

Since both AKT and c-Met cascades are essential for cell proliferation in HCC development, we investigated whether celastrol was efficient in suppressing the proliferation-associated proteins. PCNA, a proliferation-associated protein that responded during cell proliferation,^18^ was evaluated using a western blot assay. As expected, celastrol significantly reduced the protein expression of PCNA in AKT/c-Met mice, compared to the model group (Fig. 2C and D). Immunohistochemical evaluation of the ratio of cells for the nuclear antigen Ki67 has been one of the most widely used strategies for determining cell survival and chemosensitivity in tumor biological research.^19^ As expected, in the IHC data for Ki-67 staining, highly proliferative liver tumor cells were repressed upon celastrol administration, with significant reduction of the proliferation index) from tumors in AKT/c-Met mice (Fig. 3A and C). The result indicates that the suppression of the development of AKT/c-Met HCC by celastrol might potentially result from the inhibition of cell proliferation.

Fatty acid synthase (FASN) is an essential lipogenic enzyme catalyzing the ultimate process in the *de novo* biosynthesis of palmitate from acetyl–CoA and malonyl–CoA, and is frequently found in elevated levels in numerous cancers, including hepatocarcinoma.^20^ AKT/mTORC1 is supposed to be a trigger of FASN-mediated lipogenesis during carcinogenesis. Thus, we determined whether the suppressing effects of celastrol on AKT/c-Met induced HCC was involved in FASN expression. We found that histopathological alterations in all AKT/c-Met mice with a tumor burden by 7 weeks post injection were consistently accompanied by an elevation of FASN expression, compared to the WT group. In striking contrast, celastrol administration obviously attenuated AKT/c-Met induced FASN accumulation (Fig. 3B). Altogether, our data indicate that celastrol effectively inhibits AKT/c-Met induced HCC development, mainly due to suppression of cell proliferation and FASN-induced steatosis.

### Celastrol inhibits phosphorylation of AKT and ERK1/2 in the liver of AKT/c-Met mice

**Fig. 4:**
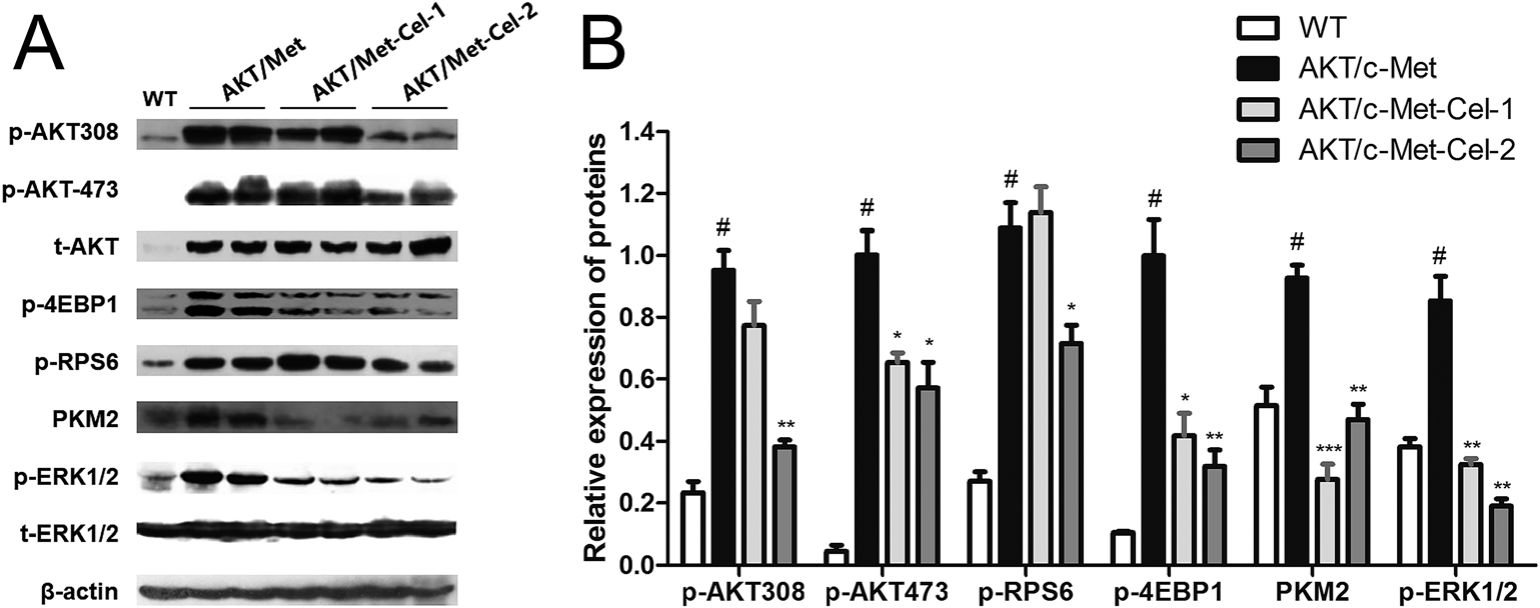
Celastrol inhibits the AKT downstream signaling transduction and suppresses the phosphorylation of ERK1/2 in the livers of AKT/c-Met HCC mice. Liver samples from each group were employed for the western blot assay, and representative bands are shown in (A). (B) Histograms show the relative protein expressions of phosphor-AKT (Thr308), phosphor-AKT (Ser473), phospho-S6 ribosomal protein (p-RPS6, Ser235/236), PKM2 (D78A4) and phospho-P44/42 MAPK (ERK1/2) (Thr202/Tyr204) levels quantified using western blot optical analysis. The housekeeping gene β-actin was applied as an internal reference. Data are expressed as mean ± sd. *n* = 5/group. #*p* < 0.05 *vs.* wt group; *p < 0.05; **p < 0.01; ***p < 0.001 *vs.* AKT/c-Met group. AKT/c-Met-Cel-1 and AKT/c-Met-Cel-2 represent tumor burden groups with 1 or 2 mg kg^−1^ celastrol, respectively.

To elucidate the molecular mechanisms underlying the therapeutic efficacy of celastrol, we evaluated the protein expression of AKT and c-Met signaling pathways in the vehicle or celastrol-administered tumors. We found that celastrol could inhibit AKT phosphorylation at regulatory residues Thr-308 and Ser-473. Moreover, down-regulation of the AKT downstream factors, phosphorylated 4E binding protein one (p-4EBP1), phosphorylated ribosomal protein S6 (p-RPS6) and pyruvate kinase M2 (PKM2) (involved in *de novo* glycolysis) were increased by celastrol (2 mg kg^−1^) treatment (Fig. 4A and B). Of note, the phosphorylation of ERK1/2 was also suppressed by celastrol (Fig. 4A and B), suggesting that the feedback deactivation of the Ras/MAPK pathway emerges after AKT signaling inhibition by celastrol (Fig. 4A and B). Therefore, celastrol retards disease progression in AKT/c-Met HCC probably by suppressing AKT and ERK1/2 phosphorylation.

## Discussion

Recently, cumulative evidence has suggested that agents derived from natural products have therapeutic effects on liver metabolic and neoplastic diseases,^21^ suggesting that searching for small molecule anti-cancer drugs from natural sources is a potential approach for preventing and controlling HCC. Undeniably, the previous preclinical studies focusing on the curative effect of celastrol on HCC models are quite persuasive. However, the evidence surrounding celastrol on HCC *in vivo* is mainly from xenografted HCC or orthotopic HCC patient-derived xenograft models *in vivo*.^22^ Therefore, whether celastrol could suppress HCC by targeting tumorigenesis from the normal liver is still not well illustrated. In fact, optional targets for treating HCC in a DEN-induced model are mainly confined to inflammatory response pathways (*e.g.*, toll-like receptor 4 and IL-6).^23^ Hence, a rapid HCC model established by hydrodynamic transfection of activated forms of AKT (myr-AKT) and c-Met (V5-c-Met) into the FVB mouse liver is employed here for evaluating the effects of celastrol on the process of HCC formation. In the present study, celastrol histologically and pathologically alleviates AKT/c-Met induced HCC. Moreover, celastrol potently inhibits the expression of proliferation-related proteins and attenuates intracellular lipid accumulation by reducing the activity of FASN. Crucially, the current study has also suggested that AKT and ERK might be potential therapeutic targets for treating HCC using celastrol.

With the synergistic effect of AKT and c-Met, rapid liver tumor development with hepatocytic differentiation has been observed as our previous study described.^24^ To investigate whether celastrol has oncotherapeutic effects in an earlier phase of tumor development, the mice were intraperitoneally treated daily with either vehicle control or celastrol (1.0 and 2.0 mg kg^−1^), 4 weeks after the hydrodynamic transfection had been finished. At the end of the experiment, it was satisfactory to observe that HCC in one (still with HA-AKT and V5-c-Met overexpression, data not shown) 2 mg celastrol-administered AKT/c-Met mouse became invisible and the mouse instead appeared healthy with a normal liver size. After daily therapy for 3 weeks, the liver weight and liver/body weight ratio, which were greatly raised in vehicle-controlled mice, were both reduced by celastrol treatment. Moreover, the preliminary findings were subsequently confirmed by H&E staining, demonstrating that celastrol is significantly capable of inhibiting foci formation in the livers of AKT/c-Met HCC mice.

The expression of proliferation-associated proteins reflects the effects of chemotherapeutic agents on cancer progression. AFP is commonly nearly undetectable in normal adult tissue and organs but is strongly expressed and secreted in proliferating hepatocytes or, specifically, HCC cells. Thus, AFP has been seen as a frequently-used diagnostic and prognostic tumor marker.^25^ Our results showed that celastrol is effective for lowering the mature AFP levels in serum, which contributes to the suppressing effect of celastrol on AFP mRNA transcription in the liver. In fact, AFP also functions as a moderator participating in several pathways involved in promoting cell proliferation and anti-apoptosis during the processes of tumorigenesis. Using the MTT assay, it was found in a previous study that the proliferation of HCC cells was consolidated by AFP protein treatment *in vitro*.^26^ Moreover, the high serum level of AFP with high liver cell proliferative activity is associated with high rates of hepatocellular carcinoma in cirrhotic patients, further suggesting that cell proliferation occurring with high AFP secretion is essential for HCC development.^27^ In this study, the protein expressions of PCNA and Ki67 in HCC tissues in AKT/c-Met mice with high serum AFP levels were significantly decreased by celastrol (2 mg) treatment. Overall, the data suggest that celastrol suppresses HCC development in AKT/c-Met HCC by arresting cell proliferation, and the effects could be involved in down-regulation of AFP mRNA translation and protein synthesis.

Glucose and lipid metabolic disorders in the liver are not only the hepatic manifestations of metabolic syndrome, but also the initial symptoms of liver cirrhosis and tumorigenesis. In our previous study, we claimed that elevation of FASN activity and its driven lipogenesis is characteristic and indispensable in AKT-induced hepatic carcinogenesis, which could be synergistically enhanced by c-Met overexpression. Here, the immune-histochemical study showed that celastrol treatment could decrease the expression of FASN in the liver tissues of AKT/c-Met HCC mice. Interestingly, a well-known previous study on celastrol has shown that celastrol acts as a powerful leptin sensitizer for anti-obesity therapy by reducing ER stress conditions.^28^ In other words, celastrol could be considered as a catalyzer for ‘fat combustion’ in obesity in the above study. In contrast, our data suggest that celastrol might be a scavenger for superfluous lipids in the liver. Therefore, celastrol is effective for inhibiting FASN-mediated liver steatosis in AKT/c-Met HCC mice, which is beneficial for preventing HCC, and so it might be considered as a therapeutic agent for metabolic diseases.

To further reveal the potential mechanisms at the molecular level underlying the anti-proliferative and lipid-lowering efficacy of celastrol, we assessed the protein expression of AKT and c-Met signaling pathways in the vehicle or celastrol-treated liver specimens. In fact, accelerating hepatic cell proliferation and invasion by activation of AKT has been extensively studied. Also, AKT de-phosphorylation could be the essential mechanism for celastrol to suppress HCC. However, the evidence surrounding the effects of celastrol on AKT in HCC was all found from the *in vitro* or *in vivo* established liver cancer model (see the Introduction section). Thus, the function of celastrol on AKT in the process of changing from normal hepatocytes to hepatoma carcinoma cells has not been adequately explained. For the first time, our data demonstrate that celastrol could repress phosphorylation of AKT at both Thr-308 and Ser-473 residues, and sequentially down-regulate AKT/mTORC1 downstream factors involved in up-regulating mRNA translation and promoting sustained cell proliferation (phospho-S6 ribosomal protein, p-S6) and glycolysis (PKM2). The phosphorylated c-Met protein subsequently uses crosstalk to trigger PI3K/AKT and mitogen-activated protein kinase (MAPK) cascades.^29^ However, it was found that celastrol has no influence on c-Met activation (Tyr 1234) (data not shown). Indeed, c-Met is a kind of membrane receptor triggering carcinogenesis by tyrosine kinase domain phosphorylation in the juxtamembrane region while it is overexpressed or combined with HGF.^6^ Therefore, it is possible to explain that there is no efficacy for celastrol binding to mature c-Met at this intracellular segment. These interesting results regarding AKT and c-Met prompt us to further investigate the effect of celastrol on the phosphorylation of ERK1/2, which is dually regulated by either AKT^30^ or c-Met.^31^ In fact, there is mounting evidence that the overactive ERK/MAPK signaling pathway in the progression of HCC could expedite cell proliferation and invasion.^32^ Surprisingly, excessive phosphorylation of ERK1/2 by co-activation of AKT/c-Met in this study was markedly repressed by celastrol, indicating that ERK could be considered as another potential target of celastrol to suppress cell proliferation in AKT/c-Met HCC mice.

Medicinal materials used in ethnomedicine such as Traditional Chinese Medicine (TCM) are a potential source of leading agents for modern clinical applications (*e.g.*, antimalarial quinine). Even so, the route from natural compounds in traditional medicines to modern drugs with pronounced therapeutic effects is always long and winding. Although the *in vitro* anti-proliferative activities of celastrol have been found in multifarious cancer cell lines, the direct targets of celastrol are still complicated.^33^ Based on the ‘target-precise’ AKT/c-Met HCC model in mice, the suppressing efficiency of celastrol on a novel AKT/FASN cascade is proven. Hence, more pre-clinical studies and systematic human studies applying celastrol or its other derivatives to alleviate the aberrant fatty acid synthesis in the liver tissues of HCCs should be scheduled.

Altogether, we conclude that celastrol could suppress AKT activation in AKT/c-Met HCC mice, which in turn down-regulates the phosphorylation of ERK1/2, but has no effect on c-Met. Down-regulation of p-AKT and p-ERK1/2 by celastrol resulted in inhibition of HCC cell proliferation by suppressing the expression of PCNA. Celastrol also reduces the activity of FASN and alleviates hepatic steatosis during HCC development. These data provide the novel *in vivo* evidence that celastrol could be potentially considered as an efficient agent for HCC prevention and therapy.

## Funding information

Research project of Hubei Provincial Department of Education (2017), grant/award number: Q20172001.

## Conflicts of interest

There are no conflicts to declare.

## List of abbreviations

HCC: Hepatocellular carcinoma
AKT: v-akt murine thymoma viral oncogene homolog
PI3K: Phosphoinositide-3 kinase
HGF: Hepatocyte growth factor
MAPK: Mitogen-activated protein kinase
NF-κB: Nuclear factor κB
SB: Sleeping beauty transposase
AFP: Alpha-fetoprotein
H&E: Hematoxylin and eosin
PBS: Phosphate-buffered saline
qPCR: Quantitative real-time polymerase chain reaction
cDNA: Complementary DNA
FASN: Fatty acid synthase
4-EBP1: 4E binding protein one
RPS6: Ribosomal protein S6
PKM2: Pyruvate kinase M2
ERK: Extracellular signal-regulated kinase

## Supplementary Material

RA-008-C8RA00522B-s001
