# Supplementary material for: Celastrol delays hepatic steatosis and carcinogenesis in a rapid AKT/c-Met-transfected hepatocellular carcinoma model via suppressing fatty acid synthase expression and AKT/ERK phosphorylation
Source: RSC Adv. 2018 Apr 16;8(25):13976–83. doi: 10.1039/c8ra00522b (PMC9079895; doi:10.1039/c8ra00522b)

Electronic Supplementary Material (ESI) for RSC Advances.
This journal is © The Royal Society of Chemistry 2018

## Supplementary materials

**Figure S1** Macroscopic appearance and H&E staining (Scale bar: 100 μm) of AKT/c-Met mouse livers at 4 weeks.

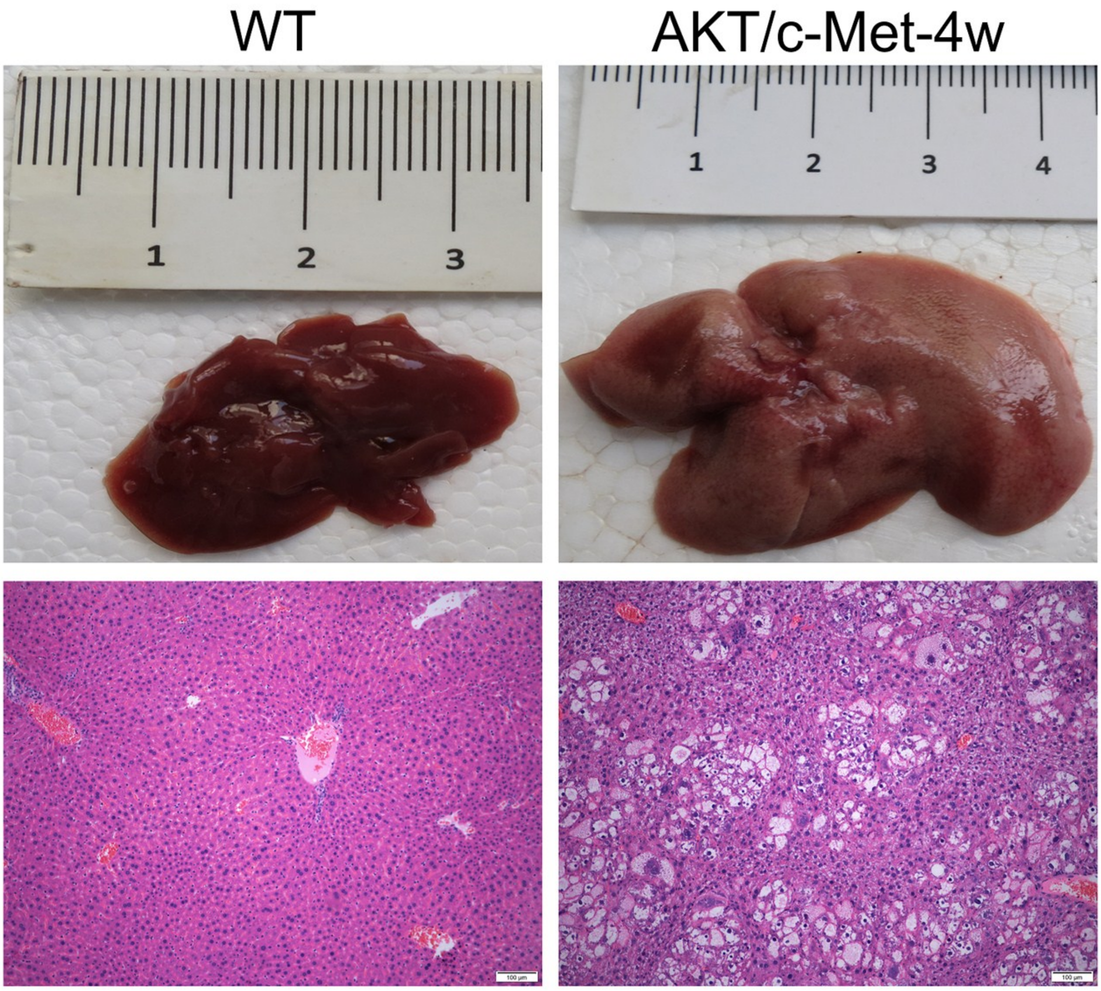

**Figure S2** No changes in liver weight were detected in Sorafenib (100 mg/kg) treated AKT/c-Met HCC. Sorafenib (100 mg/kg) or vehicle (70 % Cremophor/ethanol 3:1, 20 % PBS, and 10 % DMSO) was intraperitoneally injected daily for 3 weeks starting 4 weeks after AKT/c-Met construct injection for early stage AKT/c-Met HCC treatment.

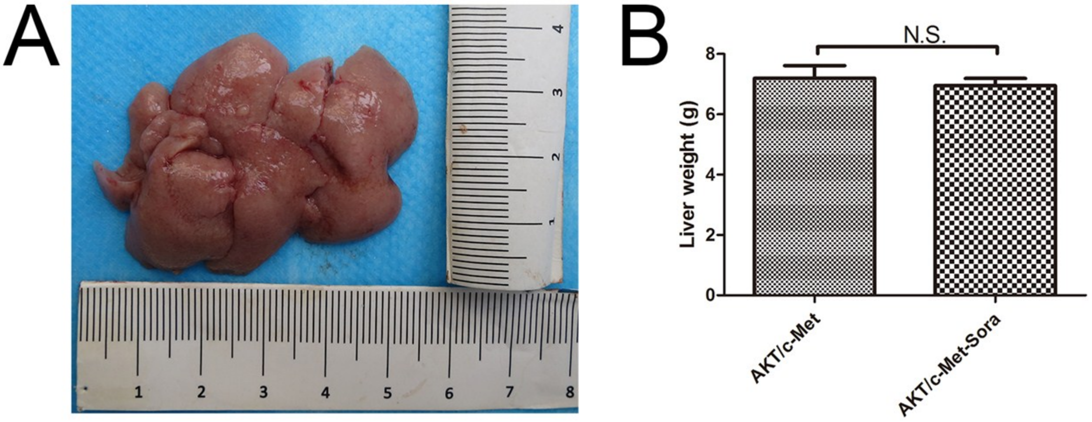

Supplement: RA-008-C8RA00522B-s001 [file RA-008-C8RA00522B-s001.pdf]
